# Anti-Obesity Effects of Collagen Peptide Derived from Skate (*Raja kenojei*) Skin Through Regulation of Lipid Metabolism

**DOI:** 10.3390/md16090306

**Published:** 2018-08-30

**Authors:** Minji Woo, Yeong Ok Song, Keon-Hee Kang, Jeong Sook Noh

**Affiliations:** 1 Department of Food Science and Nutrition and Kimchi Research Institute, Pusan National University, Busan 46241, Korea; woo07140@pusan.ac.kr (M.W.); yosong@pusan.ac.kr (Y.O.S.); 2 Yeongsan Skate Co., Ltd., Busan 48520, Korea; skate1438@naver.com; 3 Department of Food Science & Nutrition, Tongmyong University, Busan 48520, Korea

**Keywords:** collagen peptide, skate skin, high fat diet, fatty acid metabolism, cholesterol metabolism

## Abstract

This study investigated the anti-obesity effects of collagen peptide derived from skate skin on lipid metabolism in high-fat diet (HFD)-fed mice. All C57BL6/J male mice were fed a HFD with 60% kcal fat except for mice in the normal group which were fed a chow diet. The collagen-fed groups received collagen peptide (1050 Da) orally (100, 200, or 300 mg/kg body weight per day) by gavage, whereas the normal and control groups were given water (*n* = 9 per group). The body weight gain and visceral adipose tissue weight were lower in the collagen-fed groups than in the control group (*p* < 0.05). Plasma and hepatic lipid levels were significantly reduced by downregulating the hepatic protein expression levels for fatty acid synthesis (sterol regulatory element binding protein-1 (SREBP-1), fatty acid synthase (FAS), and acetyl-CoA carboxylase (ACC)) and cholesterol synthesis (SREBP-2 and 3-hydroxy-3-methylglutaryl-CoA reductase (HMGCR)) and upregulating those for β-oxidation (peroxisome proliferator-activated receptor alpha (PPAR-α) and carnitine palmitoyltransferase 1 (CPT1)) and synthesis of bile acid (cytochrome P450 family 7 subfamily A member 1 (CYP7A1)) (*p* < 0.05). In the collagen-fed groups, the hepatic protein expression level of phosphorylated 5′ adenosine monophosphate-activated protein kinase (p-AMPK) and plasma adiponectin levels were higher, and the leptin level was lower (*p* < 0.05). Histological analysis revealed that collagen treatment suppressed hepatic lipid accumulation and reduced the lipid droplet size in the adipose tissue. These effects were increased in a dose-dependent manner. The findings indicated that skate collagen peptide has anti-obesity effects through suppression of fat accumulation and regulation of lipid metabolism.

## 1. Introduction

Obesity is characterized by an abnormal accumulation of body fat that contributes to the etiologies of various metabolic disorders including dyslipidemia, hepatic steatosis, insulin resistance, and type 2 diabetes mellitus [1]. Obese individuals have high central adiposity due to the accumulation of visceral adipose tissue, which may be linked to a significantly increased risk of hepatic steatosis. The increased flux of non-esterified free fatty acid (NEFA) from the visceral fat to the liver is one of the suggested underlying mechanisms [2]. In addition, hyperlipidemia is induced by the dysregulation of hepatic lipid metabolism, which upregulates the synthesis of triglyceride (TG) and cholesterol and downregulates fatty acid oxidation [1]. These metabolic reactions could accelerate fat accumulation in the liver and exacerbate hepatic steatosis. Therefore, dietary approaches for attenuating hyperlipidemia, reducing free fatty acid levels, and inhibiting hepatic lipid synthesis and fat accumulation have attracted interest in obesity prevention or treatment.

Collagen, a fibrous protein composed of amino acid sequence glycine (Gly)-proline (Pro)-X and Gly-hydroxyproline (Hyp)-X, plays a vital role in the maintenance of the structure of various tissues and organs in the body [3]. Collagen has been widely used as a material in the food, cosmetic, and pharmaceutical industries due to its biological and functional properties [4]. Recently, marine collagen has been preferred over cattle or porcine collagen, because of bovine spongiform encephalopathy and transmissible spongiform encephalopathy, or religious reasons [5]. The skin, scale, cartilage and bone of marine fish are good sources of collagen [6]. These parts are by-products obtained during the processing of marine fish, which are considered as disposed waste [7]. Several studies have focused on the development of a technique to utilize marine collagen peptides to reduce pollution. Marine collagen has various beneficial properties such as antioxidative [8,9,10], anti-skin aging [11], antihypertensive [12,13], anti-ulcer [14], and bone integrity maintenance [15] effects. Especially as a biomaterial in tissue engineering, marine collagen has less cross-linking and higher solubility than bovine collagen, and exerts anti-ageing and anti-wrinkling effects [16].

To extract collagen peptides, several marine species have been used including red snapper [3], tuna [4], jelly fish [8], tilapia [10], salmon [17], cuttlefish [18], flatfish [19], pufferfish [20], bamboo shark [21], cod [22], carp [23], catfish [24], paper nautilus [25], marine sponges [26] and skate [27]. In particular, skate (*Raja kenojei*) is a popular food consumed in South Korea. As a result, large amounts of skate skin are disposed of as waste. Our recent study showed the lipid-lowering effect of skate skin-derived collagen peptide in genetic obese mice [28]. However, there is limited information on the effects of marine collagen in a diet-induced obese animal model. In this study, the anti-obesity effects of skate collagen peptide on improving lipid metabolism in high-fat diet (HFD)-induced obese mice were investigated. In addition, three different doses were used to examine the dose-dependent effects. Also, to elucidate the mechanism of its action with regard to synthesis and oxidation of fatty acid, adenosine monophosphate-activated protein kinase (AMPK) activation was investigated in the liver.

## 2. Results

### 2.1. Effect of Skate Collagen Peptide on Body Weight Gain and Changes in Adipose Tissue Weight and Size

**Figure 1:**
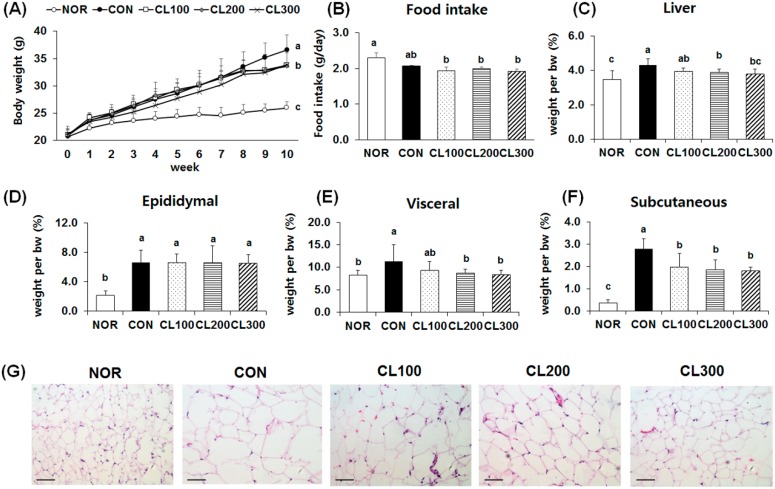
Effects of skate collagen peptide on body weight, food intake, and organ weight and histological analysis of adipose tissue in high-fat diet-fed C57BL6/J mice for eight weeks. Data are mean ± standard deviation (SD) (*n* = 9 per group). Normal (NOR) C57BL/6J mice fed a chow diet with water; control (CON) C57BL/6J mice fed a high fat diet (HFD) with water; collagen 100 (CL100), collagen 200 (CL200), and collagen 300 (CL300) C57BL/6J mice fed a HFD with oral administration of skate collagen peptide at a concentration of 100, 200, and 300 mg/kg body weight per day, respectively. ^a–c^ Different letters mean significant differences to one-way analysis of variance (ANOVA), followed by Duncan’s multiple-range test at *p* < 0.05. (**A**) change in body weight (bw) for 10 week; (**B**) food intake; (**C**) liver weight per bw; (**D**) epididymal adipose tissue weight per bw; (**E**) visceral adipose tissue weight per bw; (**F**) subcutaneous adipose tissue weight per bw; (**G**) hematoxylin and eosin staining, magnification: 200×, bar: 50 μm.

As shown in Figure 1A, there were no significant differences in the initial body weight among the experimental groups. However, HFD intake for eight weeks significantly increased body weight (*p* < 0.05). As a result, the final body weight was the highest in the control group (CON, 36.6 ± 1.0 g) followed by the 100 mg/kg collagen-fed group (CL100, 34.2 ± 0.8 g), 200 mg/kg collagen-fed group (CL200, 33.6 ± 1.1 g), 300 mg/kg collagen-fed group (CL300, 33.3 ± 1.0 g), and normal group (NOR, 26.0 ± 0.4 g) (*p* < 0.05). Among HFD-fed mice groups, collagen intake did not affect the amount of daily food intake (Figure 1B). The increased liver weight following HFD intake was reduced by collagen treatment; however, the decrease was not significant (Figure 1C). Adipose tissue weights were higher in the HFD-fed groups (Figure 1D–F). The weights of liver, visceral and subcutaneous adipose tissue in the collagen-fed groups were significantly lower compared with that in the CON group (*p* < 0.05). However, the epididymis adipose tissue was not significantly different among the HFD-fed groups. Histological analysis of the adipose tissue revealed that HFD intake facilitated the differentiation and enlargement of adipocytes. The lipid droplet size was smaller in the collagen fed-groups than in the CON group.

### 2.2. Effect of Skate Collagen Peptide on Lipid Levels in the Plasma and Hepatic Tissue

**Figure 2:**
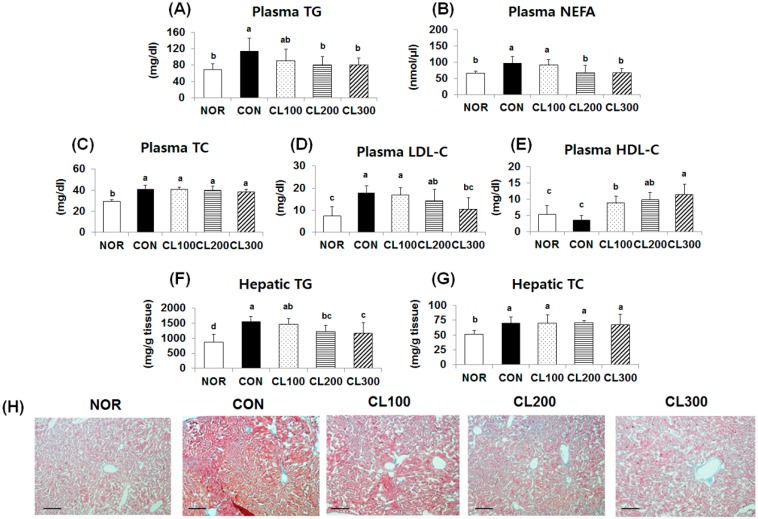
Effects of skate collagen peptide on plasma and hepatic lipid levels and histological analysis of liver tissue in high-fat diet-fed C57BL6/J mice for eight weeks. Data are mean ± SD (*n* = 9 per group). See the legend of Figure 1 for experimental groups in detail. ^a–d^ Different letters mean significant differences according to one-way ANOVA, followed by Duncan’s multiple-range test at *p* < 0.05. (**A**) plasma TG (triacylglycerol); (**B**) plasma NEFA (non-esterified free fatty acid); (**C**) plasma TC (total cholesterol); (**D**) plasma LDL-C (low-density lipoprotein cholesterol); (**E**) plasma HDL-C (high-density lipoprotein cholesterol); (**F**) hepatic TG; (**G**) hepatic TC; (**H**) Oil red O staining, magnification: 100×, bar: 100 μm.

Plasma lipid levels were higher in HFD-fed groups and were reduced by collagen intake (Figure 2A–E). Plasma TG (Figure 2A) and NEFA (Figure 2B) levels were significantly lower in the CL200 (30% and 30%, respectively) and CL300 (30% and 31%, respectively) groups compared with the levels in the CON group (*p* < 0.05). Plasma total cholesterol (TC) level was also lower; however, there was no significant difference in the HFD-fed groups (Figure 2C). Plasma low-density lipoprotein cholesterol (LDL-C) level was significantly lower in the CL200 and CL300 groups by 20% and 42%, respectively (Figure 2D, *p* < 0.05). In contrast, plasma high-density lipoprotein cholesterol (HDL-C) level was higher in the CL100, CL200, and CL300 groups by 245%, 276%, and 320%, respectively (Figure 2E, *p* < 0.05). Hepatic TG and TC levels were higher in the HFD-fed groups and were reduced by collagen intake (Figure 2F,G). In comparison with hepatic TG level in the CON group, the level was significantly lower in the CL200 and CL300 groups by 22% and 25%, respectively (Figure 2(F), *p* < 0.05). However, hepatic TC level was not significantly different between the CON and collagen-fed groups (Figure 2G). Histological analysis of the liver tissue revealed that lipid accumulation was increased by HFD intake and was suppressed by collagen intake. In particular, the degree of lipid accumulation in the CL200 and CL300 groups was similar to that in the NOR group. The histological results were in agreement with the changes in plasma and hepatic TG levels.

### 2.3. Effect of Skate Collagen Peptide on β-Oxidation in the Liver

**Figure 3:**
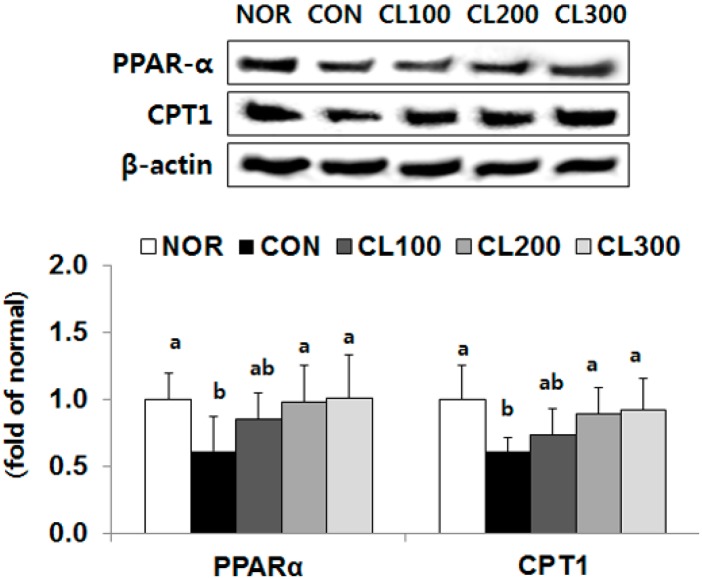
Effects of skate collagen peptide on hepatic protein expression for β-oxidation in high-fat diet-fed C57BL6/J mice for eight weeks. Data are mean ± SD (*n* = 9 per group). See the legend of Figure 1 for experimental groups in detail. ^a,b^ Different letters mean significant differences according to one-way ANOVA, followed by Duncan’s multiple-range test at *p* < 0.05. PPARα, peroxisome proliferator-activated receptor alpha; CPT1, carnitine palmitoyltransferase 1.

The protein expression levels of peroxisome proliferator-activated receptor alpha (PPAR-α) and carnitine palmitoyltransferase 1 (CPT1) (proteins involved in β-oxidation) were significantly higher in the CL200 (159% and 163%, respectively) and CL300 (146% and 151%, respectively) groups compared with their expression levels in the CON group (*p* < 0.05) (Figure 3).

### 2.4. Effect of Skate Collagen Peptide on Fatty Acid Synthesis in the Liver

**Figure 4:**
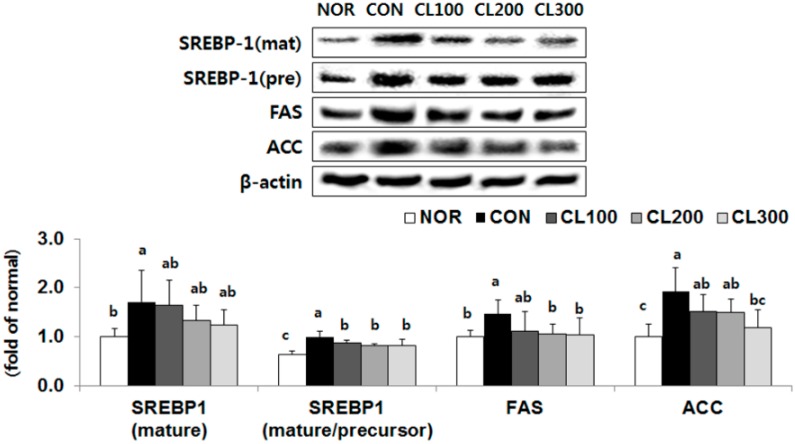
Effects of skate collagen peptide on hepatic protein expression for fatty acid synthesis in high-fat diet-fed C57BL6/J mice for eight weeks. Data are mean ± SD (*n* = 9 per group). See the legend of Figure 1 for experimental groups in detail. ^a–c^ Different letters mean significant differences according to one-way ANOVA, followed by Duncan’s multiple-range test at *p* < 0.05. SREBP-1, sterol regulatory element binding protein-1; FAS, fatty acid synthase; ACC, acetyl-CoA carboxylase.

The protein expression level of sterol regulatory element binding protein-1 (SREBP-1) (mature/precursor), a transcription factor for fatty acid synthesis, was significantly reduced in the CL100, CL200, and CL300 groups by 13%, 18%, and 18%, respectively, compared with its expression level in the CON group (Figure 4, *p* < 0.05). The protein expression level of fatty acid synthase (FAS) was significantly lower in the CL200 and CL300 groups by 28% and 29%, respectively (*p* < 0.05). In addition, the protein expression level of acetyl-CoA carboxylase (ACC) was significantly reduced in the CL300 group by 39% (*p* < 0.05).

### 2.5. Effect of Skate Collagen Peptide on Cholesterol Metabolism in the Liver

**Figure 5:**
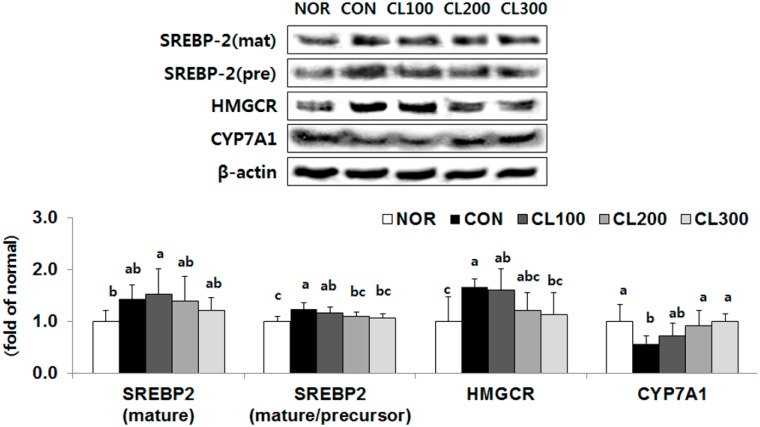
Effects of skate collagen peptide on hepatic protein expression for cholesterol synthesis and export in high-fat diet-fed C57BL6/J mice for eight weeks. Data are mean ± SD (*n* = 9 per group). See the legend of Figure 1 for experimental groups in detail. ^a–c^ Different letters mean significant differences according to one-way ANOVA, followed by Duncan’s multiple-range test at *p* < 0.05. SREBP-2, sterol regulatory element binding protein-2; HMGCR, 3-hydroxy-3-methylglutaryl-CoA reductase; CYP7A1, cytochrome P450 family 7 subfamily A member 1.

The protein expression level of SREBP-2 (mature/precursor), a transcription factor for cholesterol synthesis, was significantly reduced in the CL200 and CL300 groups by 12% and 13%, respectively, compared with its expression level in the CON group (Figure 5, *p* < 0.05). The protein expression level of 3-hydroxy-3-methylglutaryl-CoA reductase (HMGCR) was significantly lower in the CL300 group by 32% (*p* < 0.05). On the other hand, the protein expression level of cytochrome P450 family 7 subfamily A member 1 (CYP7A1) was significantly reduced in the CL200 and CL300 groups by 161% and 176%, respectively (*p* < 0.05).

### 2.6. Effect of Skate Collagen Peptide on AMPK in the Liver

**Figure 6:**
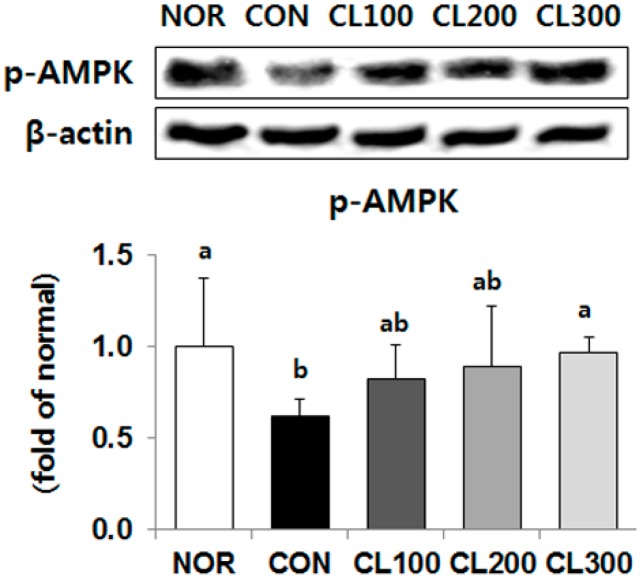
Effects of skate collagen peptide on hepatic protein expression of p-AMPK in high-fat diet-fed C57BL6/J mice for eight weeks. Data are mean ± SD (*n* = 9 per group). See the legend of Figure 1 for experimental groups in detail. ^a,b^ Different letters mean significant differences according to one-way ANOVA, followed by Duncan’s multiple-range test at *p* < 0.05. p-AMPK, phosphorylated 5′ adenosine monophosphate-activated protein kinase.

In comparison with the protein expression level of phosphorylated 5′ adenosine monophosphate-activated protein kinase (p-AMPK) in the CON group, its expression level was significantly higher in the collagen-fed groups. In the CL300 group, it was significantly higher by 156% (Figure 6, *p* < 0.05).

### 2.7. Effect of Skate Collagen Peptide on Adiponectin and Leptin Levels

Collagen intake reduced leptin levels and increased adiponectin levels in the collagen-fed groups compared with the levels in the CON group (Table 1). The adiponectin level in the CL100, CL200, and CL300 groups was higher by 110%, 123%, and 131%, respectively (*p* < 0.05). In contrast, the leptin level in the CL300 group was significantly reduced by 23% (*p* < 0.05).

## 3. Discussion

Owing to overnutrition and lifestyle changes, the prevalence of obesity has greatly increased worldwide. Researches have attempted to identify food materials or agents that can ameliorate obesity. A HFD-induced obese animal is pathophysiologically similar to an obese person [29]. As a result of the high caloric density, the consumption of a HFD causes obesity by increasing lipid levels and the adipocyte number and size [30]. Marine-derived nutrients and bioactive components have excellent potential as functional food ingredients due to their beneficial health effects [31]. Marine collagen peptides rich in glycine, glutamic acid, proline, and hydroxyproline are produced by the enzymatic hydrolysis of collagen. Among several amino acids in collagen peptides, the lipid-lowering effect of glycine has been reported [32,33]. In this study, the anti-obesity effects of collagen peptide derived from skate skin were evaluated and were found to be mediated through the regulation of hepatic lipid metabolism-related transcription factors and enzymes.

In an obese state, hyperlipidemia is closely associated with fat accumulation in major organs such as the liver and adipose tissue. In the present study, the HFD-fed groups had higher plasma TG, NFFA, and LDL-C levels and lower plasma HDL-C levels; these effects were reversed following collagen peptide administration. However, a change in TC level was not observed. The decrease in the lipid levels of collagen-fed groups might be attributed to the reduction in body weight gain and visceral and subcutaneous adipose tissue weights. Additionally, the collagen-fed groups had a lower level of hepatic TG, which was consistent with liver histological results. The TG-lowering effect of collagen suppressed adipose tissue differentiation, as demonstrated by the histological analysis of the adipose tissue. In comparison with CON mice, collagen-fed mice had smaller adipocytes. Our results were consistent with those of a previous study, in which the concentration of TG, TC, and LDL-C in HFD-fed rats was reduced by supplementation with marine collagen peptides [34]. Similarly, the intake of collagen derived from salmon [35], flathead mullet [36], and skate [28] could decrease plasma lipid levels in animals. Lipid-lowering effects were also observed in a human study showing that marine collagen peptides reduced the level of TG, free fatty acid, TC, and LDL-C, and increased that of HDL-C [37]. The intake of gelatin, a mixture of water-soluble protein derived from collagen, was reported to markedly reduce serum TG and TC levels in mice [38]. These effects might be associated with the properties of amino acid-rich collagen. A previous study found a negative correlation between plasma TG and the levels of hydroxyproline, glycine, and proline in collagen [35]. In particular, glycine intake was reported to decrease plasma free fatty acid and adipose cell size in sucrose-fed rats [32]. These results suggest that collagen peptides rich in glycine may exert hypolipidemic effects in the plasma and liver.

Abnormal fat accumulation is caused by an imbalance between lipid synthesis (lipogenesis) and breakdown (lipolysis or β-oxidation). Lipogenesis is transcriptionally regulated by SREBP-1, which controls the lipogenic enzymes FAS and ACC [39]. On one hand, PPAR-α is a transcription factor that facilitates fatty acid oxidation by upregulating target genes such as CPT1 [40]. In the current study, the hepatic protein expression levels of SREBP-1, ACC, and FAS (involved in fatty acid synthesis) in the collagen-fed groups were suppressed compared with those in the CON group. On the other hand, β-oxidation was enhanced in the collagen-fed groups by upregulating the PPAR-α and CPT1 levels. These results were consistent with those of a previous study showing that the intake of collagen peptide decreased fatty acid synthesis and increased β-oxidation in the liver of *db*/*db* mice [28]. Similarly, tuna-derived peptide was found to decrease the expression levels of SREBP-1, FAS, and ACC in differentiated 3T3-L1 adipocytes [41]. It is possible that glycine-rich collagen has a regulatory effect on some factors related to storage and energy burning, such as PPAR-α, -γ, -δ, and uncoupling protein type 2 [33]. Our results suggested that supplementation with skate collagen peptide effectively attenuated hepatic fat accumulation by improving fatty acid metabolism through the inhibition of fatty acid synthesis and facilitation of β-oxidation in the liver of HFD-fed mice.

AMPK has emerged as a regulator of energy balance that affects whole-body fuel utilization. AMPK can induce fatty acid oxidation and inhibit the synthesis of hepatic fatty acid, cholesterol and adipocyte differentiation [42]. A previous study showed that AMPK activation could ameliorate lipogenesis in the liver of mice by suppressing SREBP-1 and -2, inhibiting their target enzyme expression [43]. In contrast, the inhibition of AMPK could increase the accumulation of hepatocellular lipids in hepatocytes [44]. Moreover, AMPK is involved in the regulation of adipokines such as adiponectin and leptin, which can stimulate the phosphorylation of AMPK [45]. In obesity-induced animals, decreased adiponectin levels and increased leptin levels in the plasma have been observed [46]. However, after weight reduction, these effects were reversed with the augmentation of AMPK activation. AMPK is an important metabolic regulator; thus, it is recognized as a key target for obesity prevention. In the present study, the intake of collagen peptide increased the hepatic protein expression of p-AMPK. Furthermore, adiponectin and leptin levels were increased and decreased, respectively, in the plasma. In a previous study, the serum adiponectin level of patients with type 2 diabetes was increased following treatment with marine collagen peptides for three months compared with that of healthy control patients [37]. Furthermore, glycine treatment was reported to decrease leptin and increase adiponectin in 3T3-L1 adipocytes [33,47]. Therefore, AMPK activation and adipokine regulation by skate collagen peptide might reduce lipid accumulation through the inhibition of lipid synthesis and activation of energy production in the liver.

The reduction in plasma lipid level following the intake of fish collagen peptides is closely associated with the amino acids in the peptides. According to a previous study, the peptides in protein hydrolysates have different biological effects and physicochemical properties depending on the molecular weight or structure of the amino acids [35]. The structure and molecular weight of collagen peptides vary according to the type, source, and preparation method of the collagen [35]. The production of low molecular weight fragments is easier using collagen from marine sources than from land vertebrates [48]. Nevertheless, further research is required to study the health benefits of marine collagens with different molecular weights obtained via ultrafiltration. In conclusion, our findings revealed that the intake of collagen peptide of skate skin might exert anti-obesity activities through reduction of body weight gain and visceral adipose tissue, and improve the dyslipidemia via regulation of hepatic lipid metabolism and activation of AMPK, as well as its targeted adiponectin.

## 4. Materials and Methods

### 4.1. Animals and Diets

Male C57BL6/J mice (5 weeks old) were purchased from Orient, Inc. (Seongnam, Korea). The mice were raised under controlled temperature (23 ± 1 °C) and humidity (50 ± 5%) conditions with a 12 h light-dark cycle. After a 1 week acclimation period, the mice were divided into five groups (*n* = 9 per group) based on body weight as follows; (1) normal group (NOR), given AIN-76A chow diet and water as vehicle by gavage; (2) control group (CON), given HFD and water as vehicle by gavage; (3) CL100, given HFD and 100 mg/kg body weight (bw)/day of skate collagen peptide by gavage; (4) CL200, given HFD and 200 mg/kg bw/day of skate collagen peptide by gavage; CL300, given HFD and 300 mg/kg bw/day of skate collagen peptide by gavage. The dosage given to the mice was converted from a human equivalent dosage: assuming the human equivalent dose for 1.0 g/60 kg/day × 12.3 = 0.2 g/kg/day. A conversion coefficient of 2.3 was used to account for differences between mice and humans [49]. To examine the dose-dependent effects, three different doses were determined for oral administration based on a previous study [30]. HFD with 60% kcal fat was provided from Central Lab Animal Inc. (Seoul, Korea) which has been commonly used for the development of obesity in experimental rodent models [50]. Skate collagen peptide was dissolved in water and orally administered to mice. The collagen peptide was obtained from Yeongsan Skate Co., Ltd. (Jeollanam-do, Korea) with an average molecular weight of 1050 Da. The amino acid composition of the collagen sample used in this study was as follows: glycine 22.09%, glutamate 10.78%, proline 9.02%, alanine 7.66%, arginine 7.84%, aspartate 7.11%, hydroxyproline 6.85%, serine 5.71%, lysine 3.49%, leucine 3.67%, threonine 3.42%, valine 3.34%, isoleucine 2.45%, phenylalanine 2.23%, methionine 2.15%, histidine 1.38%, and others 0.81%. The mice had free access to the diet and water. The dietary intake was checked daily and body weight was measured every week. After 8 weeks, all mice were fasted for 12 h and sacrificed after CO_2_ anesthetization. Blood was obtained using heparin tubes from the heart and the organs were collected after perfusion with ice-cold phosphate-buffered saline (PBS, 10 mM, pH 7.2). Epididymis adipose tissue was derived from the fat attached to the two testicles of the mice. Visceral adipose tissue was excised from the perirenal fat depot. Subcutaneous adipose tissue was collected from the fat located beneath the skin of the legs. The organs were stored at −80 °C until use. The study was approved by the Pusan National University Institutional Animal Care and Use Committee (Approval number: PNU-2016-1640).

### 4.2. Plasma Lipid, Aminotransferase, and Adipokines Levels

The levels of plasma TG, TC, and HDL-C were measured using commercially available kits (AM157S-K, AM202-K, and AM203-K; Asan Pharmaceutical Co., Seoul, Korea). NEFA was determined using commercial kits (ab65341; Abcam Inc., Cambridge, MA, USA). Plasma LDL-C level was calculated using a previously reported method [51] In addition, commercial kits were used to evaluate adipokines such as leptin (#ADI-900-019A; Enzo Life Sciences AG, Lausen, Switzerland) and adiponectin (LF-EK0239; AbFrontier, Seoul, Korea).

### 4.3. Hepatic Lipid Concentration

The hepatic lipids of the liver homogenate were extracted according to a modified method [52]. In brief, liver tissue was homogenized in PBS and extracted using chloroform and methanol (2:1, *v*/*v*). The extracts were vortexed for 2 h, filtered, and dried. Hepatic TG and TC levels were measured with the same commercial kit used for measuring plasma lipid levels.

### 4.4. Western Blot Analysis

Quantitation of protein was carried out by Western blot assay as previously described [53]. In brief, protein was separated by sodium dodecylsulfate polyacrylamide gel and transferred to a nitrocellulose membrane (Amersham Biosciences, Uppsla, Sweden). The targeted protein band was detected using CAS-400 (Core Bio, Seoul, Korea). The calculation was performed using ImageJ software (National Institutes of Health, Bethesda, MD, USA). Protein expression was normalized to that of β-actin. The primary antibodies used in this study were β-actin (ab8227) and FAS (ab22759), which were purchased from Abcam Inc. (Cambridge, UK). Phospho-AMPKα (p-AMPK, #2535) was obtained from Cell Signaling Technology (Beverly, MA, USA). SREBP-1 (sc-8984), ACC (sc-26817), PPAR-α (sc-9000), CPT1 (sc-139482), SREBP-2 (sc-5603), HMGCR (sc-33827), and CYP7A1 (sc-25536) were purchased from Santa Cruz Biotechnology (Santa Cruz, CA, USA). The secondary horseradish peroxidase-conjugated antibodies (from Abcam Inc.) were donkey anti-rabbit IgG H&L (ab6802), rabbit anti-goat IgG H&L (ab6741), and rabbit anti-mouse IgG H&L (ab6728).

### 4.5. Histological Analysis

The liver and adipose tissue were fixed in 4% formalin for preparation of frozen and paraffin blocks, respectively. Sections of the frozen-blocked liver tissues were cut at a thickness of 3 µm using a microtome (CM1510S-3; Leica, Wetzlar, Germany) and stained with Oil Red O. Sections of the paraffin-blocked adipose tissue were cut using a microtome at a thickness of 3 µm (Microm HM 325; Thermo Fisher Scientific, Waltham, MA, USA) and stained with hematoxylin and eosin. The slides were examined under an optical microscope (Nikon ECLIPSE Ti; Nikon Corp., Tokyo, Japan).

### 4.6. Statistical Analysis

Data are presented as the mean ± SD. Statistical analysis was performed using SPSS version 23 (SPSS Inc., Chicago, IL, USA). The significance of differences were determined by one-way analysis of variance (ANOVA) followed by Duncan’s multiple-range test. Differences with *p* < 0.05 were considered significant.

## Figures and Tables

**Table 1:** Changes in leptin and adiponectin levels of high-fat diet-fed C57BL6/J mice for eight weeks.

Group ^(1)^	Leptin	Adiponectin
NOR	54.6 ± 5.0 ^c^	198.6 ± 14.1 ^d^
CON	122.6 ± 34.9 ^a^	214.4 ± 46.3 ^c,d^
CL100	98.0 ± 24.6 ^a,b^	236.3 ± 21.5 ^b,c^
CL200	97.0 ± 22.9 ^a,b^	263.8 ± 35.3 ^a,b^
CL300	94.0 ± 15.2 ^b^	281.1 ± 17.9 ^a^

Data are mean ± SD (*n* = 9 per group). ^(1)^ See the legend of Figure 1 for experimental groups in detail. ^a–d^ Different letters mean significant differences according to one-way ANOVA, followed by Duncan’s multiple-range test at *p* < 0.05.
